# Molecular and serological biomarkers to predict trastuzumab responsiveness in HER-2 positive breast cancer

**DOI:** 10.25122/jml-2023-0163

**Published:** 2023-11

**Authors:** Noor Abdullah, Loma Al-Mansouri, Naael Ali, Najah Rayish Hadi

**Affiliations:** 1 Department of Pharmacology, College of Medicine, University of Basrah, Basrah, Iraq; 2 Department of Medicine, College of Medicine, University of Basrah, Basrah, Iraq; 3 Department of Microbiology, College of Medicine, University of Basrah, Basrah, Iraq; 4 Department of Pharmacology and Therapeutics, Faculty of Pharmacy, University of Kufa, Najaf, Iraq

**Keywords:** CTLA-4, T lymphocyte, HER-2 positive breast cancer, insulin-like growth factor-I, trastuzumab

## Abstract

HER-2-positive breast cancer is characterized by its aggressive nature, poor prognosis, and reduced overall survival. The emergence of trastuzumab resistance is currently considered a global problem. The immune system plays a pivotal role in tumor progression and development. Cytotoxic T lymphocyte-associated protein-4 (CTLA-4) and other immune checkpoint proteins may be potential prognostic factors and therapeutic targets for breast cancer. This study aimed to determine the correlation between CTLA-4 expression in peripheral blood and insulin-like growth factor-1 (IGF-1) serum levels and their impact on trastuzumab responsiveness in HER-2-positive patients with breast cancer. CTLA-4 expression was analyzed in peripheral blood cells using quantitative PCR, while IGF-1 serum levels were assessed through electrochemiluminescence assays. There was a significant increase in CTLA-4 expression at cycle 9, which continued to increase until it reached 4.6 at cycle 17. High IGF-1 levels were observed in newly diagnosed HER-2 positive patients before trastuzumab therapy, significantly decreasing post-therapy (p=0.001). Co-targeting HER-2 and IGF-1 receptors may reduce the risk of recurrence and improve outcomes. In addition, targeted CTLA-4 molecules may improve patient survival and prevent recurrence.

## INTRODUCTION

Breast cancer is one of the most common cancers in women worldwide. Age, heredity, reproductive status, physical inactivity, and obesity are all common risk factors for breast cancer [1]. Approximately 20% of new breast cancer cases are HER-2 positive, often associated with poor prognosis and higher aggressiveness compared to HER-2 negative types [2]. Since the 1990s, trastuzumab, a monoclonal antibody targeting the extracellular domain of the HER-2 receptor, in combination with chemotherapy, has been the gold standard in breast cancer treatment [3]. Trastuzumab (Herceptin) has improved outcomes in patients with HER-2-positive breast cancer, both in early and advanced stages [4]. However, the emergence of trastuzumab resistance is currently considered a global problem. Despite a year-long trastuzumab treatment (17 cycles), over 20% of patients experience recurrence or metastasis, demonstrating the complexity and heterogeneity of the tumor [5]. Unfortunately, there are no currently available noninvasive biomarkers that can accurately detect a patient's responsiveness to trastuzumab [6]. Finding new treatments to overcome trastuzumab treatment failure is essential to improve mortality in HER-2-positive metastatic breast cancer [7, 8]. A great challenge is identifying individuals who have a high risk of recurrence. Genetic testing provides valuable prognostic information for early-stage patients with breast cancer in order to obtain possible prognostic information as part of their treatment regimens [9]. Gene expression profiles and whole genome sequencing are increasingly being incorporated into the treatment planning process for breast cancer. Using genetic approaches can reduce costs and potential harm due to cancer therapy, especially for patients who do not respond to treatment [10]. The objective of this study was to determine the correlation between cytotoxic T lymphocyte-associated protein-4 (CTLA-4) expression in peripheral blood and insulin-like growth factor-1 (IGF-1) serum level as biomarkers for predicting trastuzumab treatment efficacy in HER-2 positive breast cancer. CTLA-4 (CD152) is a cell surface receptor that inhibits T lymphocyte proliferation and function and suppresses the immune response to tumors [11]. Expression of CTLA-4 enables cancer cells to evade antitumor T cell responses and may reduce the anti-cancer immune response, making tumors more likely to spread. Similarly, it would be anticipated that natural T-regs, which constitutively express CTLA-4, would interact with residual B7 molecules more effectively than responder T cells, resulting in T-cell inhibition rather than proliferation [12]. In the emerging era of immunotherapy, CTLA-4 expression in breast cancer is a potential clinical marker and a rational therapeutic target [13]. Trastuzumab is a recombinant humanized monoclonal antibody that works by binding to the protein's extracellular domain [14]. When used in combination with chemotherapy, it significantly extends patient survival by delaying disease progression [15]. One of its mechanisms involves the activation of tyrosine kinase, leading to the internalization and degradation of the HER-2 receptor [16]. Trastuzumab also induces tumor cell lysis through antibody-dependent cellular cytotoxicity (ADCC), which is particularly effective in cases of HER-2 overexpression [17]. Many factors, such as IGF-1, transforming growth factor, platelet-derived growth factor, hepatocyte growth factor, fibroblast growth factor, and vascular endothelial growth factor, promote cell proliferation and tissue repair [18]. Trastuzumab-resistant cells have been more likely to exhibit elevated IGF-1R signaling [19]. Increased signaling via IGF-1R has been involved in a reduced response to trastuzumab in breast cancer cells in vitro [20]. In recurrent breast cancer, IGF-1R has been shown to heterodimerize with HER-2 [21, 22]. Elevated levels of IGF-1 are associated with an increased risk of both the development and recurrence of breast cancer, primarily due to its roles in myogenesis and anti-apoptotic activities. The co-inhibition of HER-2 and IGF-1R inhibits the proliferation of HER-2+ breast cancer cells. Specifically, trastuzumab-resistant breast cancer cells exhibit crosstalk between IGF-1R and HER-2 signaling since IGF-1R physically interacts with HER-2.

This study aimed to explore the potential of CTLA-4 and IGF-1 as biomarkers for predicting the efficacy of trastuzumab treatment in HER-2-positive breast cancer. This involved analyzing the gene expression of CTLA-4 and serum levels IGF-1 in peripheral blood cells and sera to detect the tumor's responsiveness to the targeted therapy trastuzumab.

## MATERIAL AND METHODS

### Study design and participants

This longitudinal descriptive observational study followed patients for one year, covering 17 cycles of trastuzumab treatment. Patients were newly diagnosed, aged over 20 years, non-pregnant, and with no history of cardiac problems or chronic diseases. Participants were recruited from Basrah Oncology Center, and all patients were confirmed as HER-2 positive by immunohistochemistry before receiving chemotherapy, radiation, or immunotherapy. Age, parity, menstrual history, family history of breast cancer, grade and stage of tumors, body mass index (BMI), and smoking history were included in the questionnaire.

### Sample collection

Five milliliters of venous blood were drawn from each participant using aseptic techniques. The samples were divided into two parts: one in ethylenediaminetetraacetic acid (EDTA) for RNA extraction and CTLA-4 expression analysis in peripheral blood cells via real-time polymerase chain reaction (PCR), and the other in a gel tube for serum IGF-1 level measurement.

### RNA extraction and analysis

RNA was extracted from blood samples collected at baseline (before trastuzumab treatment), after 9 cycles, and at the end of the 17-cycle trastuzumab therapy, using the GENOM kit protocol for whole blood. The purified RNA was frozen at -70 degrees Celsius using a deep freezer (Nuaire, Japan). The amount and quality of extracted RNA were measured with a spectrophotometer (NanoDrop) by observing its absorption at 260 and 280 nanometers. The AccuPower^®^ PCR PreMix dNTPs kit (Bioneer, Korea) was used to convert 5 µg of total RNA into cDNA. Gene-specific primers were used to amplify the mRNA expression of CTLA-4 and beta-actin. The primers were purchased from Macrogen Company and Alpha DNA, Canada (Table 1).

**Table 1:** Primer sequences for CTLA-4 and Beta-Actin (β-actin)

Gene	Primer sequence
CTLA-4 Forward CTLA-4 Reverse β-actin Forward β-actin Reverse	CTTCAGTCACCTGGCTGTCA CTCAGCTGAACCTGGCTACC GGACTTCGAGCAAGAGATGG AGCACTGTGTTGGCGTACAG

### Real-time PCR data analysis

Beta-actin was used as an internal control (housekeeping gene) for real-time PCR. Calculations were made to determine the mRNA expression of CTLA-4 in proportion to beta-actin and the intensity of the fluorescence signals for these genes. Results were expressed as fold changes in CTLA-4 expression relative to beta-actin.

### Serum insulin-like growth factor-I assay

IGF-1 levels were measured using electrochemiluminescence (ECL) assays (cobas e) in samples taken at baseline and after 17 cycles of trastuzumab therapy. The normal value for IGF-1 is age-dependent. The average serum concentration lies somewhere between 174 and 355 ng/ml.

### Statistical analysis

All analyses were performed using SPSS software version 24.0. The normality of the variables was determined using the Shapiro–Wilk test. Any non-parametric variables were analyzed using the Kruskal–Wallis test; otherwise, one-way ANOVA was used for parametric data. Statistically significant results were considered if the p value was equal to or less than 0.05. The Livak method (Delta-Delta Ct) was used for qRT-PCR data analysis.

## RESULTS

A total of 50 patients were enrolled in the study. Among them, 60% were younger than 50 years, while the remaining 40% were 50 years or older. Regarding menopausal status, 48% were premenopausal. The majority (68%) had children, and six participants were single (not married). All patients were non-smokers, and 18% reported a family history of breast cancer (Table 2).

**Table 2:** Demographic characteristics of patients

Factors	No.	%
**Age** Age <50 Age ≥50	30 20	60 40
**Menopausal status** Premenopausal Menopausal	24 26	48 52
**Parity status** Pluriparous Nulliparous Not married	34 10 6	68 20 12
**Family history of breast cancer** Positive Negative	9 41	18 82
**Smoking history** Non-smoker Smoker	50 0	50 0

According to tumor-node-metastasis (TNM) staging of breast cancer, 4% of patients were in stage I, 54% were in stage II, and 42% were in stage III. Out of all tumors, 50% were classified as grade II, while the other half were classified as grade III. Thirty-three of the patients received the ACT chemotherapy protocol (Adriamycin, Cyclophosphamide, and Taxol), whereas 17 (34% of the total) received the ACD chemotherapy protocol (Adriamycin, Cyclophosphamide, and Docetaxel). Only 18% of patients had a BMI within the normal range, 34% were overweight, and the largest percentage, 48%, were categorized as obese. Twelve percent of participants in this research developed recurrence after one year of trastuzumab treatment (Table 3).

**Table 3:** Clinical and histopathological features of patients

Variables	No.	%
**TNM stage** I II III	2 27 21	4 54 42
**Tumor grade** Grade 2 Grade 3	25 25	50 50
**Estrogen receptor status** Positive Negative	9 41	18 82
**Progesterone receptor status** Positive Negative	9 41	18 82
**Chemotherapy protocol** ACT ACD	33 17	66 34
**Recurrence post-trastuzumab treatment** Yes No	6 44	12 88

TNM: tumor-node-metastasis; ACT: adriamycin, cyclophosphamide, and taxol; ACD: adriamycin, cyclophosphamide, and docetaxe

### Fold change expression of CTLA-4

**Figure 1:**
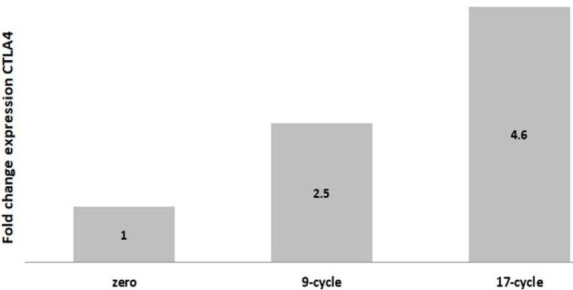
CTLA-4-fold change in peripheral mononuclear cells among patients with HER-2 positive breast cancer during Trastuzumab therapy

The gene expression of CTLA-4 mRNA was quantitatively analyzed using real-time qPCR. We employed a relative expression assay and used the ΔΔCT (Delta Cycle Threshold) method to calculate the fold change in expression. The β -Actin gene served as the internal control (housekeeping gene), and its value was subtracted to normalize the results. The expression level of CTLA-4 in the control sample (taken at cycle 1 of the treatment) was set as the baseline (normalized to 1). The expression levels of CTLA-4 showed a significant increase during the course of treatment. By the 9th cycle, the expression had risen to 2.5 times the baseline level, and it continued to increase until it reached 4.6 at cycle 17 (Figure 1).

CTLA-4 mRNA levels in the total RNA samples were quantified using RT-qPCR and normalized against β-Actin levels. Analysis showed CTLA-4 mRNA upregulation, increasing 2.5-fold by cycle 9 and 4.6-fold by cycle 17. A significant increase in CTLA-4 expression was observed at cycle 17 in patients with both recurrent and non-recurrent breast cancer post-trastuzumab therapy (p-value<0.05) (Table 4).

**Table 4:** CTLA-4 expression fold change in patients with and without breast cancer recurrence

Recurrence status		Fold change at baseline	Fold change at cycle 9	Fold change at cycle 17
Yes	N	6	6	6
	Mean	1	1.44	6.65
No	N	44	44	44
	Mean	1	2.55	4.01
p-value*		0.601	0.541	0.05

**Figure 2:**
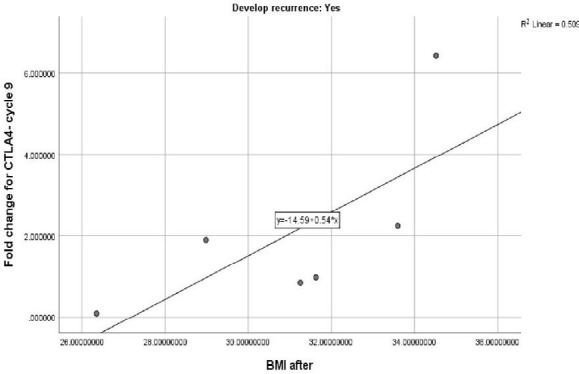
Correlation between CTLA-4 at cycle 9 and BMI in patients with recurrence

IGF-1 levels were high among newly diagnosed HER-2 positive patients before starting trastuzumab therapy (normal value: 174-335 ng/ml). However, after the completion of trastuzumab therapy, a statistically significant reduction in serum IGF-1 levels was observed (p-value=0.001) (Table 3). The median IGF-1 values were 416 and 401 ng/ml before and after trastuzumab therapy, respectively, indicating significantly high levels of this growth factor among patients who later experienced recurrence. There was a significant decrease in IGF-1 level at the end of the study after completion of trastuzumab therapy in patients who did not experience recurrence (Tables 5 and 6). A positive correlation was also observed between BMI at the end of treatment and the expression of CTLA-4 at cycle 9 in patients who experienced recurrence (Figure 2).

**Table 5:** Comparison of IGF-1 levels before and after one year of Trastuzumab therapy

	IGF-1 (ng/ml) baseline	IGF-1 (ng/ml) cycle-17
No.	50	50
Median	364.75	185.8
SD	61.33	110.69
p-value	0.002	0.0001

**Table 6:** Comparison of IGF-1 levels in patients with and without recurrence

Recurrence status		IGF-1 baseline	IGF-1 at the end of Trastuzumab
Yes	N	6	6
	Median	416	401
	SD	41.06	80.6
No	N	44	44
	Median	363.65	178.4
	SD	58.17	93.17
p-value		0.003	0.0001

**Figure 3:**
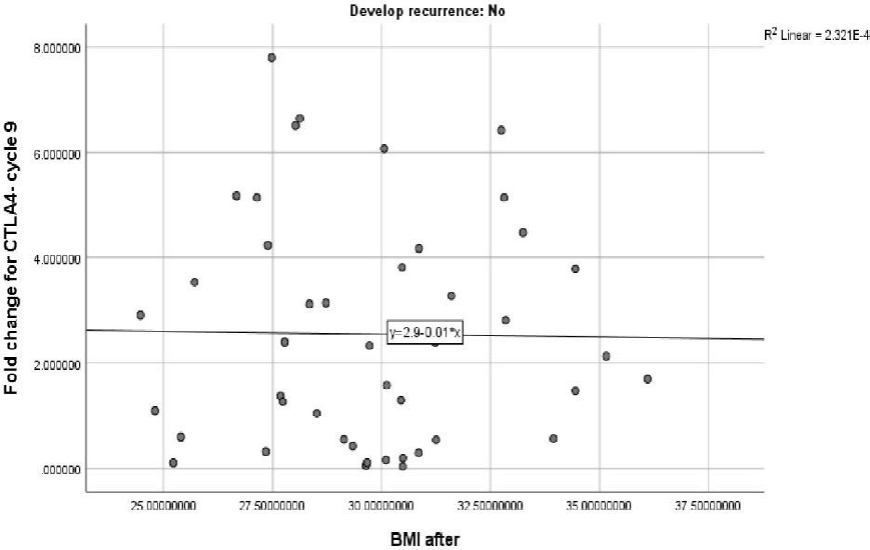
Correlation between CTLA-4 expression and BMI in patients without recurrence

There was no significant relationship between CTLA-4 expression at cycle 9 and BMI after trastuzumab therapy at the end of the study in patients without recurrence (Figure 3).

## DISCUSSION

Breast cancer is the most common form of malignancy affecting Iraqi women, accounting for one-third of all cancers [21, 22]. HER-2-positive breast cancers account for more than twenty percent of cases and have a high rate of disease recurrence and mortality [23]. Given the increasing incidence of breast cancer and HER-2 mutation, identifying new biomarkers to correlate with therapeutic response is crucial. Our study focused on evaluating the gene expression of immune checkpoint genes in the peripheral blood and investigating the relationship with the clinical status of patients with breast cancer. Patients' ages were analyzed across 50 people who received trastuzumab therapy for a year. The findings align with the observation that HER-2-positive cancers tend to be more prevalent in women under the age of 40 [24]. We observed a significant up-regulation in the gene expression of CTLA-4, which became evident as early as cycle 9, with a 2.5-fold increase and continued to increase throughout the trastuzumab therapy, reaching a peak of 4.6 at cycle 17. CTLA-4 is a key inhibitory immune checkpoint molecule normally expressed at low levels on the surface of naive T cells and T regulatory cells (T-Reg). However, CTLA-4 expression can be up-regulated in response to T-cell receptor stimulation.

Such stimulation is triggered by different molecules, such as kinases, phosphatases, and phospholipases, which account for the expression of immune checkpoint molecules in these effector cells (CD8, CD4, and T-Reg) [25]. The finding implies that in response to trastuzumab, expression of CTLA-4 in peripheral cells may be important in modulating patients' immune responses and cancer defenses. During the follow-up period, the expression of CTLA-4 was significantly higher among patients who developed recurrence at the end of therapy (p-value=0.05). Up-regulated gene expression of CTLA-4 on T cells, as well as interaction with its ligand on T cells (T-regs), leads to decreased T cell proliferation and functional activity [26]. We also found a positive correlation between patients' BMIs after trastuzumab treatment and their CTLA-4 expression at cycle 9. This suggests that overweight individuals may have an increased CTLA-4 expression, which in turn inhibits T-cell activation and increases the risk of trastuzumab non-responsiveness and tumor recurrence [27]. Prolonged antigen exposure, which is common in cancer, can lead to tolerized T cells via CTLA-4 expression. In addition, as CTLA-4 has both intrinsic and extrinsic routes of action, it inhibits cell cycle and interleukin-2 (IL-2) production, resulting in immune response blockade [28]. In fact, increased expression of CTLA-4 is associated with diminished immune defense against the tumor.

CTLA-4 is involved in both the intrinsic and extrinsic pathways during its impact on the cell cycle and its influence on the cell stimulation for interleukin-2 production, thereby leading to the control of immune responses [29]. An increase in inhibitory gene expression may be due to tumor overactivity during this treatment cycle, as inhibitory genes suppress the normal immune system against cancer cells. This could be used to anticipate future recurrences and unresponsiveness. Hence, checkpoint genes could be suggested as biomarkers and therapeutic targets for treating breast cancer. Monoclonal antibodies (Ipilimumab and Tremelimumab) that target CTLA-4 are among the most widely used therapeutic agents in oncology at the moment. Anti-CTLA-4 antibodies substantially increase the immune system's ability to suppress tumor growth and improve the prognoses of patients with cancer when used alone or in combination with other therapeutic agents [30]. Blocking the expression of CTLA-4 in HER-2-positive patients with breast cancer may benefit the treatment. To our knowledge, no previous study has shown the role of immune checkpoint protein (CTLA-4) expression in peripheral blood among HER-2-positive patients. The observed fluctuations in fold change expression of CTLA-4 may be attributed to various factors, including environmental influences, dietary habits, and exposure to inter-individual variables that can impact gene expression. CTLA-4 expression may reflect higher activity of T-Reg cells in patients with breast cancer, which may play a role in tumor establishment and development [31, 32]. The activity of T-Reg cells during the course of trastuzumab may carry a risk of recurrence in the future, and this is supported by the high expression level of CTLA-4 in patients who developed recurrence.

High IGF-1R expression or phosphorylation levels in tumor samples were found to correlate with lower response rates to trastuzumab-based bio-chemotherapy in HER-2+ breast cancer. Our study found that the level of IGF-1 was significantly higher in patients with recurrence, while there was a significant reduction in patients who completed trastuzumab therapy without tumor recurrence. IGF-I serum biomarkers are most commonly used to evaluate anti-HER-2 therapy in patients with HER-2-positive metastatic breast cancer. In addition, these biomarkers are also useful for predicting the therapeutic response and monitoring HER-2-targeted therapy in patients with HER-2-positive metastatic breast cancer.

## CONCLUSION

CTLA-4 expression could be used as a potential biomarker for patients who may experience recurrence or resistance to trastuzumab. Our preliminary findings suggest that a combined therapeutic approach, targeting both HER-2 and the insulin-like growth factor-1 and its receptors, could potentially reduce recurrence risks and enhance treatment outcomes. Additionally, the strategic use of targeted CTLA-4 molecules might significantly improve patient survival rates and prevent recurrence. Subsequently, the concomitant administration of a CTLA-4 blocker with trastuzumab may improve patient outcomes.

## References

[1] Holm JB, Rosendahl AH, Borgquist S (2021). Local biomarkers involved in the interplay between obesity and breast cancer. Cancers (Basel).

[2] Loibl S, Gianni L (2017). HER2-positive breast cancer. Lancet.

[3] Brown KA (2021). Metabolic pathways in obesity-related breast cancer. Nat Rev Endocrinol.

[4] Singh DD, Lee H-J, Yadav DK (2022). Clinical updates on tyrosine kinase inhibitors in HER2-positive breast cancer. Front Pharmacol.

[5] Ma B, Ma Q, Wang H, Zhang G (2016). Clinical efficacy and safety of T-DM1 for patients with HER2-positive breast cancer. Onco Targets Ther.

[6] Song PN, Mansur A, Lu Y, Della Manna D (2022). Modulation of the Tumor Microenvironment with Trastuzumab Enables Radiosensitization in HER2+ Breast Cancer. Cancers (Basel).

[7] Bloom MJ, Song PN, Virostko J, Yankeelov TE, Sorace AG (2022). Quantifying the Effects of Combination Trastuzumab and Radiation Therapy in Human Epidermal Growth Factor Receptor 2-Positive Breast Cancer. Cancers (Basel).

[8] Syed AK, Woodall R, Whisenant JG, Yankeelov TE, Sorace AG (2019). Characterizing Trastuzumab-Induced Alterations in Intratumoral Heterogeneity with Quantitative Imaging and Immunohistochemistry in HER2+ Breast Cancer. Neoplasia.

[9] Zhou ZR, Yang ZZ, Yu XL, Guo XM (2018). Highlights on molecular targets for radiosensitization of breast cancer cells: Current research status and prospects. Cancer Med.

[10] Pondé N, Brandão M, El-Hachem G, Werbrouck E, Piccart M (2018). Treatment of advanced HER2-positive breast cancer: 2018 and beyond. Cancer Treat Rev.

[11] Carlino F, Diana A, Ventriglia A, Piccolo A (2022). HER2-Low Status Does Not Affect Survival Outcomes of Patients with Metastatic Breast Cancer (MBC) Undergoing First-Line Treatment with Endocrine Therapy plus Palbociclib: Results of a Multicenter, Retrospective Cohort Study. Cancers (Basel).

[12] Sajjadi E, Guerini-Rocco E, De Camilli E, Pala O (2023). Pathological identification of HER2-low breast cancer: Tips, tricks, and troubleshooting for the optimal test. Front Mol Biosci.

[13] Sajjadi E, Venetis K, Ivanova M, Fusco N (2022). Improving HER2 testing reproducibility in HER2-low breast cancer. Cancer Drug Resist.

[14] Zhang M, Li B, Liao H, Chen Z (2022). Targeting HER3 or MEK overcomes acquired Trastuzumab resistance in HER2-positive gastric cancer-derived xenograft. Cell Death Discov.

[15] Hua X, Bi XW, Zhao JL, Shi YX (2022). Trastuzumab Plus endocrine therapy or chemotherapy as first-line treatment for patients with hormone receptor–positive and HER2-positive metastatic breast cancer (SYSUCC-002). Clin Cancer Res.

[16] Kern R, Panis C (2021). CTLA-4 expression and its clinical significance in breast cancer. Arch Immunol Ther Exp (Warsz).

[17] Li YC, Zhou Q, Song QK, Wang RB (2020). Overexpression of an Immune Checkpoint (CD155) in Breast Cancer Associated with Prognostic Significance and Exhausted Tumor-Infiltrating Lymphocytes: A Cohort Study. J Immunol Res.

[18] Li CJ, Lin LT, Hou MF, Chu PY (2021). PD-L1/PD-1 blockade in breast cancer: The immunotherapy era (Review). Oncol Rep.

[19] Carosella ED, Ploussard G, LeMaoult J, Desgrandchamps F (2015). A Systematic Review of Immunotherapy in Urologic Cancer: Evolving Roles for Targeting of CTLA-4, PD-1/PD-L1, and HLA-G. Eur Urol.

[20] Sinha M, Zhang L, Subudhi S, Chen B (2021). Pre-existing immune status associated with response to combination of sipuleucel-T and ipilimumab in patients with metastatic castration-resistant prostate cancer. J Immunother Cancer.

[21] Pérez-Ruiz E, Melero I, Kopecka J, Sarmento-Ribeiro AB (2020). Cancer immunotherapy resistance based on immune checkpoints inhibitors: Targets, biomarkers, and remedies. Drug Resist Updat.

[22] Feng D, Wang J, Shi X, Li D (2023). Membrane tension-mediated stiff and soft tumor subtypes closely associated with prognosis for prostate cancer patients. Eur J Med Res.

[23] Vardas V, Tolios A, Christopoulou A, Georgoulias V (2023). Immune Checkpoint and EMT-Related Molecules in Circulating Tumor Cells (CTCs) from Triple Negative Breast Cancer Patients and Their Clinical Impact. Cancers (Basel).

[24] da Silva JC, Scandolara TB, Kern R, Jaques HDS (2022). Occupational Exposure to Pesticides Affects Pivotal Immunologic Anti-Tumor Responses in Breast Cancer Women from the Intermediate Risk of Recurrence and Death. Cancers (Basel).

[25] Zhang Z, Fang T, Lv Y (2022). A novel lactate metabolism-related signature predicts prognosis and tumor immune microenvironment of breast cancer. Front Genet.

[26] Sanabria-Figueroa E, Donnelly SM, Foy KC, Buss MC (2015). Insulin-like growth factor-1 receptor signaling increases the invasive potential of human epidermal growth factor receptor 2-Overexpressing breast cancer cells via Src-focal adhesion kinase and Forkhead box protein M1. Mol Pharmacol.

[27] Coudert B, Pierga JY, Mouret-Reynier MA, Kerrou K (2014). Use of [(18)F]-FDG PET to predict response to neoadjuvant trastuzumab and docetaxel in patients with HER2-positive breast cancer, and addition of Bevacizumab to neoadjuvant trastuzumab and docetaxel in [(18)F]-FDG PET-predicted non-responders (AVATAXHER): an open-label, randomised phase 2 trial. Lancet Oncol.

[28] Nabholtz JM, Reese DM, Lindsay MA, Riva A (2002). HER2-positive breast cancer: update on Breast Cancer International Research Group trials. Clin Breast Cancer.

[29] McCormack PL (2013). Pertuzumab: a review of its use for first-line combination treatment of HER2-positive metastatic breast cancer. Drugs.

[30] Yu Y (2023). The Function of NK Cells in Tumor Metastasis and NK Cell-Based Immunotherapy. Cancers (Basel).

[31] Popović M, Silovski T, Križić M, Dedić, Plavetić N (2023). HER2 Low Breast Cancer: A New Subtype or a Trojan for Cytotoxic Drug Delivery?. Int J Mol Sci.

[32] Young AC, Quach HT, Song H, Davis EJ (2020). Impact of body composition on outcomes from anti-PD1+/− anti-CTLA-4 treatment in melanoma. J Immunother Cancer.

